# Yeast (*Saccharomyces cerevisiae*)-fermented moist feed for improving growth performance, carcass traits, and meat quality of broiler chickens

**DOI:** 10.5455/javar.2026.m1043

**Published:** 2026-06-22

**Authors:** Md. Abu Rayhan, Jesmin Aktar, Shahnur Rahman Sohag, Mohammad Al-Mamun, Md. Abul Hashem, Momota Rani Debi

**Affiliations:** 1 Department of Animal Nutrition, Bangladesh Agricultural University, Mymensingh 2202, Bangladesh; 2 Department of Animal Science, Bangladesh Agricultural University, Mymensingh 2202, Bangladesh

**Keywords:** Yeast, fermentation, moist feed, feed nutrient, broiler performance

## Abstract

**Objectives:** Despite numerous studies on *Saccharomyces cerevisiae*, most focus on single-ingredient fermentation, with limited applicability to fermented complete moist feed with gradual replacement of the control diet. Therefore, this study aimed to explore the impact of different inclusion levels of yeast-fermented moist feed on the overall productivity of broiler chicken.

**Materials and Methods:** Corn-soy-based mash feed was fermented for 48 h at 29 °C in a controlled environment with 2% yeast and 40% water. A feeding trial was conducted with 300 Indian River day-old chicks for a period of 35 days. Birds were divided into five dietary treatment groups: CD = Control Diet; YFM-25 = 75% Control + 25% yeast-fermented moist feed; YFM-50 = 50% Control + 50% yeast-fermented moist feed; YFM-75 = 25% Control + 75% yeast-fermented moist feed; YFM-100 = 100% yeast-fermented moist feed having six replications (10 birds/replication).

**Results:** Broilers fed with 75% and 100% yeast-fermented moist feed had significantly (*p* < 0.05) better growth performance, higher final body weights (1843 gm and 1860 gm), improved FCR (1.76 and 1.73), and carcass traits with increased (*p* < 0.05) dressing percentages and higher (*p* < 0.05) meat yields in certain cuts than the other treatment groups. Additionally, incorporating 100% fermented moist feed improved (*p* < 0.05) nutrient digestibility, bone mineralization, and meat quality, including higher crude protein and lower fat content (*p* < 0.05). This study also demonstrated that 100% yeast-fermented moist feed significantly (*p* < 0.05) reduced production costs and increased profit margins.

**Conclusions:** Overall, incorporating yeast-fermented moist feed, especially at 75% and 100% levels, offers an effective natural strategy to improve broiler performance, enhance meat quality, and increase economic returns.

## 1. Introduction

The poultry industry plays a crucial role in food safety and nutrition worldwide. Poultry, especially broiler chickens, serves as a vital source of meat and eggs, with no cultural or religious restrictions. But up to 70% of the total cost of commercial poultry production is attributed to broiler feed. Therefore, antibiotics have been used as feed additives to enhance growth performance and control diseases in poultry, thereby increasing profits. However, due to the negative effects of antibiotics on human health, their use as growth promoters in poultry diets is now restricted [1, 2]. As a result, there is a growing trend in the poultry industry to seek alternatives, such as feed fermentation [3, 4]. Fermentation with yeast is emerging as a viable substitute for antibiotic growth promoters [5, 6, 7]. The main objective of fermentation is to convert sugars and other carbohydrates into usable end products with higher protein content and quality, as well as other beneficial effects [5, 8].

Feeding broilers fermented moist feed may provide a more effective approach to maximizing nutrient utilization and improving efficiency compared to conventional dry feeding methods [9]. Yeast-fermented moist feed can enhance broiler performance through several mechanisms. First, the yeast cell wall contains mannan oligosaccharide (MOS), a significant probiotic that positively affects the host by modifying the microbial community and promoting intestinal villus development [10]. Second, ethanol, a byproduct of fermentation, serves as a source of energy for broilers, increasing feed utilization [11]. Third, fermentation increases protein availability and enhances its biological value by improving protein quality [12]. Fourth, it enhances the bioavailability of nutrients [13]. Finally, fermentation aids in the degradation of toxic components and antinutritional factors while producing antioxidant and antimicrobial compounds that boost disease resistance [8].

Fermenting hand-mixed feed with yeast provides the necessary nutrients for yeast cell synthesis, enhancing the fermentation process and improving overall performance [14]. A previous study demonstrated that fermentation of low-quality agro-industrial by-products significantly enhances the protein content, improves amino acid profiles, and reduces the fiber content (NDF, ADF, cellulose, and hemicellulose) in agricultural by-products, enhancing its digestibility and making it more suitable as poultry feed [15, 16, 17, 18]. Thus, the fermentation process holds significant potential for improving the quality of feed ingredients, ultimately enhancing broiler performance and health.

Among various types of yeast, *Saccharomyces cerevisiae* is commonly utilized in animal nutrition for fermentation, promoting the growth and development of broilers [19]. Fermenting rice bran with *S. cerevisiae* and urea increased protein content, reduced fiber, enhanced the growth performance, and depressed the cholesterol levels in broilers [7]. Hand-mixed rations fermented with *S. cerevisiae* increased CP and reduced CF, leading to developed BWG, better FCR, carcass yield, and bone mineralization [6].

Despite numerous studies on *S. cerevisiae*, most have focused on the fermentation of single feed ingredients, which may not fully reflect practical feeding systems. To the best of our knowledge, information on the fermentation of a complete corn–soy-based diet and its dose-dependent effects on broiler performance and meat quality remains limited. Therefore, the present study was conducted to ferment a complete hand-mixed diet using *S. cerevisiae* and to evaluate the effects of graded inclusion levels (0–100%) of yeast-fermented moist feed on growth performance, carcass yield, and meat quality in broiler chickens, with the aim of identifying an optimal and cost-effective feeding strategy.

## 2. Materials and Methods

### 2.1. Ethical approval

The present study was conducted at the Shahjalal Animal Nutrition Field Laboratory, Bangladesh Agricultural University, Mymensingh-2202, Bangladesh. Experimental procedures and bird handling were permitted by the Institutional Animal Ethics Committee (Approval no.: AWEEC/BAU/2026(2)/04(a)).

### 2.2. Preparation of broiler diet

The least-cost ration was formulated using commonly available ingredients throughout the experimental period. Active dried baker’s yeast (*Saccharomyces cerevisiae)* used in this study was procured from Angel Yeast Co., Ltd., China (*S. cerevisiae* E491, 1 × 10^10^ CFU/gm). The ingredients used and the calculated nutrient composition of the broiler diet are shown in Table 1 below. To prepare hand-mixed yeast-fermented moist feed, the ration was properly mixed with 2% yeast and 40% water and then kept under anaerobic conditions at room temperature (29 °C) for 48 h based on the results of previous studies [6]. The yeast-fermented moist feed was used to prepare the experimental diet.

**Table 1.** The ingredients used and the nutrient composition of the broiler ration fed from 7 days old to 35 days old.

Ingredients	Amount (kg)	Calculated Chemical Composition	Amount (%)
Corn	48	CP	21.98
Soybean Meal	39.5	*ME (kcal/kg)	3030.55
Rice Polish	4	CF	5.16
Oil	5	▪Ca%	0.77
Limestone	1	▪Available P%	0.679
Dicalcium Phosphate	1.5	Methionine	0.53
aVitamins Mineral premixes	0.3	Lysine	1.23
Methionine	0.07		
Lysine	0.2		
Choline Chloride	0.15		
Mycotoxin Binder	0.03		
Salt	0.25		
Total	100		

*Calculated using the formula of Wiseman and Lessire [20]; ▪According to NRC [21]. ^a^2.5 gm vitamin mineral premix: Vitamin A, 13,500 I.U; Vitamin D3, 1,500 I.U; α-DL-tocopherol acetate, 50 mg; Menadione, 2 mg; Thiamine, 3 mg; Riboflavin, 5 mg; Pyridoxine, 4 mg, Cobalamin, 15 µg; Folic acid, 200 µg; Nicotinic acid 60 mg: Calcium pantothenate, 30 mg; Cholin, 750 mg, Ascorbic acid, 150 mg.

### 2.3. Experimental birds and other management practices of the birds

The trial with broiler chicken was conducted for 35 days using 300 (Indian River) day-old broiler chicks. Chicks were obtained from Kazi Farms Group. The average weight of the DOC was 38 gm. For the first seven days of the experiment, chicks were provided with pre-starter crumble feed containing 23% CP and 2950 kcal ME/kg of diet to facilitate adaptation. After 7 days, a hand-mixed ration was prepared and supplied to the birds from day 8 to 35 (Table 1). At the last stage of the adaptation period, healthy chicks were selected, weighed, and divided into different dietary treatment groups. There were 5 dietary treatment groups, each consisting of 60 chicks, with six replicates per treatment (10 birds/replicate). The dietary treatments were formulated as follows.

CD = Control Diet (Hand-mixed dry feed without fermentation)

YFM-25 = 75% Control + 25% Yeast-fermented moist feed

YFM-50 = 50% Control + 50% Yeast-fermented moist feed

YFM-75 = 25% Control + 75% Yeast-fermented moist feed

YFM-100 = 100% Yeast-fermented moist feed

Birds were housed in a hygienic, naturally ventilated building with concrete walls. A total of 30 cages were used for the experiment, each providing a floor space of 0.91 m^2^ (120 cm × 76 cm). Before the arrival of chicks, the poultry house floors and cages were thoroughly cleaned and washed using pressurized water through a hosepipe, followed by disinfection with bleaching powder. All feeders, drinkers, containers, and other essential equipment were also properly cleaned, disinfected with phenyl solution, air-dried, and left unused for one week before chick placement. As a litter material, good quality sawdust was used and spread over the concrete floor to a depth of 5 cm. The litter material was properly stirred weekly to prevent the buildup of harmful gas (ammonia gas). A footbath was maintained at the entrance of both the poultry compound and the house to ensure biosecurity. An electric bulb was used for brooding the chicks. All birds were vaccinated against Newcastle and Gumboro diseases according to the recommended vaccination schedule. Throughout the experimental period, birds were provided an adequate amount of feed and fresh water on an *ad libitum* basis.

### 2.4. Experimental procedure and data collection

#### 2.4.1. Proximate Analysis

The proximate components and nutrient composition of the basal diets were determined according to the approaches defined by AOAC [22]. Triplicate samples were analyzed, and the results were stated as percentages. The CP content was examined with the Micro-Kjeldahl method described by Kohn and Allen [23].

#### 2.4.2. Growth performance of broiler

The preliminary body weights of the chicks were recorded at the commencement of the study using a digital weighing balance with appropriate precision (Model: HESA 3303) and subsequently measured every week to calculate body weight gain (BWG). Feed intake was determined by subtracting the amount of leftover feed from the amount offered in the previous morning. The FCR was calculated as the total feed consumed divided by the corresponding weight gain measured at the end of the starter, finisher, and overall production phases.

#### 2.4.3. Carcass evaluation

After completion of the feeding trial, one bird per replication (total 6 birds/treatment) was humanely slaughtered for carcass evaluation. To ensure empty gastrointestinal tracts, feed and drinking water were withdrawn overnight. The weights of the carcass components were then recorded.

#### 2.4.4. Meat quality

The meat quality of birds was analyzed in the laboratory of the Animal Science Department, Bangladesh Agricultural University. At first, meat samples from each group were placed in a plastic bag, sealed, chilled in a refrigerator, and then stored at 4 °C for 24 h. The meat superiority characteristics, such as pH, cooking loss, drip loss, and water-holding capacity, were evaluated.

#### 2.4.5. pH measurement

About 30 gm of meat samples were first blended and then diluted with the required amount (200 ml) of distilled water. The pH of the meat was measured using a calibrated digital pH meter (HANNA H1 2211 pH/ORP).

#### 2.4.6. Determination of breast and thigh meat color

The carcasses were refrigerated for 24 h, and the color of each breast meat sample was measured separately for each experiment with a Konica Minolta Chroma Meter CR-410 (Konica Minolta, Inc., Tokyo, Japan) according to CIE LAB [24].

#### 2.4.7. Cooking loss

Cooking loss was calculated according to Bai et al. [25]. The samples were weighed and sealed in a double-layered polythene bag. Then kept in a water bath at 80 °C for 30 min. After 30 min, samples were removed from the water bath and left to dry on the surface for 10 min. Then, the samples were weighed again. Then, the cooking loss was measured by the following formula:


\[
\mathrm{Cooking}~\mathrm{Loss}~ \left(\%\right) = \frac{\left(\mathrm{Sample}~\mathrm{weight}~\mathrm{before}~\mathrm{cooking}~ - ~\mathrm{sample}~\mathrm{weight}~\mathrm{after}~\mathrm{cooking}\right) }{\mathrm{Sample}~\mathrm{weight}~\mathrm{before}~\mathrm{cooking}} \times 100 
\]

#### 2.4.8. Drip loss

Drip loss was calculated according to Honikel [26]. Approximately 25 gm (wet weight) of uniformly shaped muscle was excised from the same location of the breast for each sample and immediately balanced to determine the initial weight. Each sample was then suspended on a string and placed in an airtight container, followed by storage at 4 °C. After 24 h, the samples were retrieved and reweighed using a digital balance to determine the final weight. Drip loss was subsequently calculated using the following formula:


\[
\mathrm{Drip}~\mathrm{Loss}~ \left(\%\right) =\frac{\left(\mathrm{Initial}~\mathrm{weight}~\mathrm{of}~\mathrm{the}~\mathrm{sample}~ - ~\mathrm{final}~\mathrm{weight}~\mathrm{of}~\mathrm{the}~\mathrm{sample}\right) }{\mathrm{Initial}~\mathrm{weight}~\mathrm{of}~\mathrm{the}~\mathrm{sample}} \times 100
\]

#### 2.4.9. Water holding capacity (WHC) (%)

The water-holding capacity (WHC) of the breast muscle was determined using a centrifugation assay [27]. Approximately 1 gm of breast muscle was cut into cubes from each replicate and placed into a centrifuge tube. The samples were then centrifuged at 3,000 × *g* at 4 °C for 10 min. WHC was calculated based on the amount of exuded water using the following formula:


\[
\mathrm{WHC}~ \left(\%\right) = \frac{\mathrm{Sample}~\mathrm{weight}~\mathrm{after}~\mathrm{centrifugation}~}{\mathrm{Sample}~\mathrm{weight}~\mathrm{before}~\mathrm{centrifugation}} \times 100
\]

#### 2.4.10. Meat composition

The nutritional composition of meat was measured according to the method described by AOAC [22] and also Kohn and Allen [23]. Equal portions of breast and thigh meat were homogenized together, and the composite sample was analyzed for meat composition.

#### 2.4.11. Nutrient digestibility

Acid-insoluble ash (AIA) was used as a marker to calculate the nutrient digestibility of the experimental diet. The birds were fed twice daily during the whole digestion trial period. A common diet (Table 1) was supplemented with 1% acid-insoluble ash to determine the nutrients’ digestibility. Each diet was given to the broiler chicks from 31 to 35 days of experimental age. Throughout the digestibility period, all feces were collected daily at feeding time. The forced-air oven (at 60 °C for 48 h) was used for drying the feces. The dried feces were then ground before nutrient analysis. The nutrient compositions of each diet are also analyzed in the laboratory. The techniques used for AIA determination were described by CIE LAB [24] and Vogtmann et al. [28]. This technique has been successfully employed to determine nutrient digestibility in non-ruminant animals.

#### 2.4.12. Tibia ash and bone mineralization

Only the right tibia was measured for weight and length, and the left tibia was used for mineral density, content, and ash. The shank length and diameter were measured by using slide calipers. Tibias were removed between the tibial-tarsal joint and the tibial-femoral joint. The tibia and shank were then autoclaved at 121 °C for 15 min, and any remaining flesh and cartilage caps were removed carefully by hand. The length and diameter at the center of the diaphysis were determined using calipers and then oven-dried at 105 °C for 24 h. The bones were then ignited at 650 °C for 13 h, and the percentage of ash was calculated by using the following formula:


\[
\mathrm{Ash}~ \left(\%\right) = \frac{~\mathrm{The}~\mathrm{ash}~\mathrm{weight}}{\mathrm{The}~\mathrm{dried}~\mathrm{bone}~\mathrm{weight}}
\]

#### 2.4.13. Cost-benefit analysis

A cost-benefit analysis was conducted by calculating the costs of chicks, feed ingredients, litter, electric bulbs, disinfectant, other equipment, and vaccines. The prices were based on the market price of all ingredients at that time. In this calculation, other costs, such as labor, electricity, and medicine, were not included.

### 2.5. Statistical analysis

Raw experimental data were recorded using Microsoft Excel. All recorded and calculated data were subsequently analyzed using IBM SPSS Statistics 22. A one-way analysis of variance (ANOVA) based on a completely randomized design (CRD) was conducted to assess the significance of treatment effects. Mean comparisons among treatments were performed using Duncan’s Multiple Range Test (DMRT). Statistical significance was set at *p* < 0.05.

## 3. Results

### 3.1. Growth performance

The growth performance of broilers after feeding different dietary experimental groups is given in Table 2. Final body weight (FBW), body weight gain (BWG), and average daily gain (ADG) were significantly increased (*p* < 0.05) with the increasing addition of yeast-fermented moist feed, and the highest value was observed in the YFM-100 group compared to the others. Feed intake (FI) did not vary significantly but was comparatively higher in the control group (3289 gm). The feed conversion ratio (FCR) was significantly improved in the YFM-100 group (*p* < 0.05) compared to the other dietary treatment groups.

**Table 2.** Growth performance of broiler chicken fed yeast-fermented moist feed under different dietary treatments.

Parameters	CD	YFM-25	YFM-50	YFM-75	YFM-100	SEM	*p*-value
FBW (gm)	1627 ± 51.6^b^	1663 ± 73.7^ab^	1733 ± 10.2^ab^	1843 ± 70.2^a^	1860 ± 60.8^a^	29.9	0.012
BWG (gm)	1589 ± 51.6^b^	1625 ± 3.7^ab^	1695 ± 110.2^ab^	1805 ± 70.2^a^	1822 ± 60.8^a^	27.9	0.012
ADG (gm)	45.41 ± 1.45^b^	46.44 ± 2.1^ab^	48.44 ± 3.16^ab^	51.58 ± 2.01^a^	52.00 ± 1.7^a^	0.856	0.012
FI (gm)	3289 ± 115.3	3151 ± 29	3140 ± 26.9	3176 ± 45.7	3136 ± 13.9	19.9	0.057
FCR	2.06 ± 0.04^a^	1.94 ± 0.07^ab^	1.85 ± 0.104^bc^	1.76 ± 0.04^bc^	1.73 ± 0.05^c^	0.037	0.001

Here, CD = control diet; YFM-25 = 75% Control + 25% Yeast-fermented moist feed; YFM-50 = 50% Control + 50% Yeast-fermented moist feed; YFM-75 = 25% Control + 75% Yeast-fermented moist feed; YFM-100 = 100% Yeast-fermented moist feed; gm = gram; FBW = final body weight; BWG = body weight gain; ADG = average daily gain; FI = feed intake; FCR = feed conversion ratio; SEM = standard error of mean; ^a, b, c,^ Means bearing uncommon superscripts in a row differ significantly.

### 3.2. Carcass characteristics

The consequence of yeast-fermented moist feed on carcass yield and several visceral organs of broilers is shown in Table 3. Here, the live weight of the bird was higher in the YFM-100 group, where the dressing yield did not significantly vary (*p* > 0.05) between the YFM-100 (68.9%) and the YFM-75 groups (68.3%). However, a significantly higher dressing yield (*p* < 0.05) was observed in these two groups compared to the control and YFM-25 groups. Head, shank, breast, thigh, and drumstick weights were significantly higher (*p* < 0.05) in the YFM-75 and YFM-100 groups than in the other treatment groups. Gizzard, heart, and spleen weights did not vary significantly (*p* > 0.05) among all dietary experimental groups.

**Table 3.** Carcass characteristics of broiler chicken fed yeast-fermented moist feed under different dietary treatments.

Parameters	CD	YFM-25	YFM-50	YFM-75		YFM-100	SEM	*p*-value
Live Weight (gm)	1630 ± 41^b^	1670 ± 62^ab^	1730 ± 111^ab^	1850 ± 74^a^		1870 ± 62^a^	29.8	0.011
Dressing Yield (%)	65.6 ± 0.56^b^	65.8 ± 0.85^b^	67.4 ± 0.58^ab^	68.3 ± 1.09^a^		68.9 ± 0.86^a^	0.40	0.002
Head (gm)	34.3 ± 1.15^b^	34.6 ± 1.52^b^	39.3 ± 3.21^ab^	41.6 ± 5.51^a^		40.6 ± 1.53^a^	1.05	0.014
Neck (gm)	41.0 ± 2.00	38.7 ± 6.11	45.7 ± 3.79	46.7 ± 3.21		45.0 ± 3.61	1.19	0.141
Shank (gm)	36.3 ± 2.08^c^	32.3 ± 3.0^abc^	40.3 ± 2.52^ab^	45.3 ± 1.53^a^		42.3 ± 3.21^ab^	1.34	0.001
Liver (gm)	33.6 ± 0.57^c^	37.0 ± 2.0^abc^	46.0 ± 1.73^ab^	39.7 ± 5.77^a^		41.7 ± 0.57^ab^	1.28	0.004
Gizzard (gm)	54.3 ± 3.21^a^	49.7 ± 2.08^a^	49.7 ± 6.43^a^	55.0 ± 9.17^a^		49.3 ± 1.53^a^	1.34	0.534
Heart (gm)	9.6 ± 0.57^a^	9.3 ± 0.57^a^	8.7 ± 0.57^a^	9.0 ± 1.00^a^		9.3 ± 1.15^a^	0.20	0.640
Breast (gm)	141 ± 6.03^b^	164 ± 23.1^ab^	184 ± 20.6^ab^	202 ± 14.47^a^		211 ± 6.56^a^	7.61	0.007
Thigh (gm)	68 ± 1.00^b^	69 ± 4.00^b^	82 ± 7.94^ab^	87 ± 5.29^a^		87 ± 1.53^a^	2.50	0.004
Spleen (gm)	1.00 ± 0.00^a^	1.33 ± 0.60^a^	1.67 ± 0.60^a^	1.33 ± 0.60^a^		1.33 ± 0.60^a^	0.13	0.655
Drumstick (gm)	70.3 ± 1.50^c^	73.7 ± 4.90^bc^	82.3 ± 4.70^abc^		87.7 ± 6.80^ab^	92.3 ± 2.50^a^	2.42	0.008

Here, CD = control diet; YFM-25 = 75% Control + 25% Yeast-fermented moist feed; YFM-50 = 50% Control + 50% Yeast-fermented moist feed;YFM-75 = 25% Control + 75% Yeast-fermented moist feed; YFM-100 = 100% Yeast-fermented moist feed; gm = gram; % = percentage; SEM = standard error of the mean; ^a, b, c,^ means bearing uncommon superscripts in a row differ significantly.

### 3.3. Meat quality

The effect of yeast-fermented moist feed on the meat quality of broilers is presented in Table 4. Cooking loss (%) and drip loss (%) were significantly highest (*p* < 0.05) in the CD group (25.8% and 3.75%, respectively) and lowest in the YFM-100 group (22.3% and 3.29%, respectively). Water-holding capacity was higher in the YFM-75 and YFM-100 groups than in the others. The meat pH values were not significantly different (*p* > 0.05) among the dietary treatment groups. However, the meat pH value was comparatively higher with an increasing level of fermented moist feed in the diet.

**Table 4.** Meat quality of broiler chicken fed yeast-fermented moist feed under different dietary treatments.

Parameters	CD	YFM-25	YFM-50	YFM-75	YFM-100	SEM	*p*-value
Cooking Loss (%)	25.8 ± 0.87^a^	24.9 ± 0.44^a^	23.3 ± 0.69^b^	22.9 ± 0.78^b^	22.3 ± 0.41^b^	0.45	< 0.001
Drip loss (%)	3.75 ± 0.10^a^	3.51 ± 0.48^ab^	3.46 ± 0.20^ab^	3.38 ± 0.06^b^	3.29 ± 0.15^b^	0.02	0.024
WHC (%)	92.5 ± 0.5	93.1 ± 0.70	94.3 ± 0.30	94.6 ± 0.56	94.2 ± 1.88	0.98	0.143
pH	6.1 ± 0.07	6.1 ± 0.04	6.3 ± 0.09	6.2 ± 0.08	6.3 ± 0.19	0.008	0.050

Here, CD = control diet; YFM-25 = 75% control + 25% Yeast-fermented moist feed; YFM-50 = 50% Control + 50% Yeast-fermented moist feed; YFM-75 = 25% Control + 75% Yeast-fermented moist feed; YFM-100 = 100% Yeast-fermented moist feed; % = percentage; WHC = water-holding capacity; SEM = standard error of the mean; ^a, b, c,^ means bearing uncommon superscripts in a row differ significantly.

### 3.4. Meat color

The effect of yeast-fermented moist feed on breast meat color and thigh meat color of broilers is displayed in Table 5. In breast and thigh meat, lightness (L*) was non-significant (*p* > 0.05) but comparatively higher in the yeast-fermented groups compared to the control. In the breast meat, redness (a*) & yellowness (b*) were significantly (*p* < 0.05) higher in the YFM-100 (6.32 & 7.53, respectively) and YFM-75 (6.28 & 6.86, respectively) groups compared to the control and other groups. In the thigh meat, the significant (*p* < 0.05) redness value was 5.53, and the yellowness value was 7.74 after feeding the complete fermented feed.

**Table 5.** Meat color of broiler chicken fed yeast-fermented moist feed under different dietary treatments.

Parameters	CD	YFM-25	YFM-50	YFM-75	YFM-100	SEM	*p*-value
Breast Meat Color							
L*	47.76 ± 0.33	48.67 ± 2.85	51.91 ± 2.49	45.93 ± 3.66	50.02 ± 0.45	0.75	0.090
a*	4.18 ± 0.20^b^	4.43 ± 0.32^b^	4.89 ± 0.21^b^	6.28 ± 0.39^a^	6.32 ± 0.66^a^	0.26	<0.001
b*	8.17 ± 0.31^a^	5.93 ± 0.28^cd^	5.33 ± 0.45^d^	6.86 ± 0.77^bc^	7.53 ± 0.43^ab^	0.29	<0.001
Parameters	CD	YFM-25	YFM-50	YFM-75	YFM-100	SEM	*p*-value
Thigh Meat Color							
L*	48.86 ± 1.31	50.90 ± 2.88	52.97 ± 1.66	52.58 ± 0.49	53.05 ± 1.71	0.58	0.070
a*	4.34 ± 0.43^b^	4.41 ± 0.08^b^	4.77 ± 0.39^ab^	4.96 ± 0.17^ab^	5.53 ± 0.37^a^	0.13	0.007
b*	8.49 ± 0.28^a^	7.85 ± 0.16^ab^	7.81 ± 0.72^ab^	6.95 ± 0.21^b^	7.74 ± 0.37^ab^	0.16	0.013

Here, CD = control diet; YFM-25 = 75% Control + 25% Yeast-fermented moist feed; YFM-50 = 50% Control + 50% Yeast-fermented moist feed; YFM-75 = 25% Control + 75% Yeast-fermented moist feed; YFM-100 = 100% Yeast-fermented moist feed; L* = Lightness; a* = Redness; b* = Yellowness; SEM = standard error of the mean; ^a, b, c,^ means bearing uncommon superscripts in a row differ significantly.

### 3.5. Meat composition

**Figure 1.**
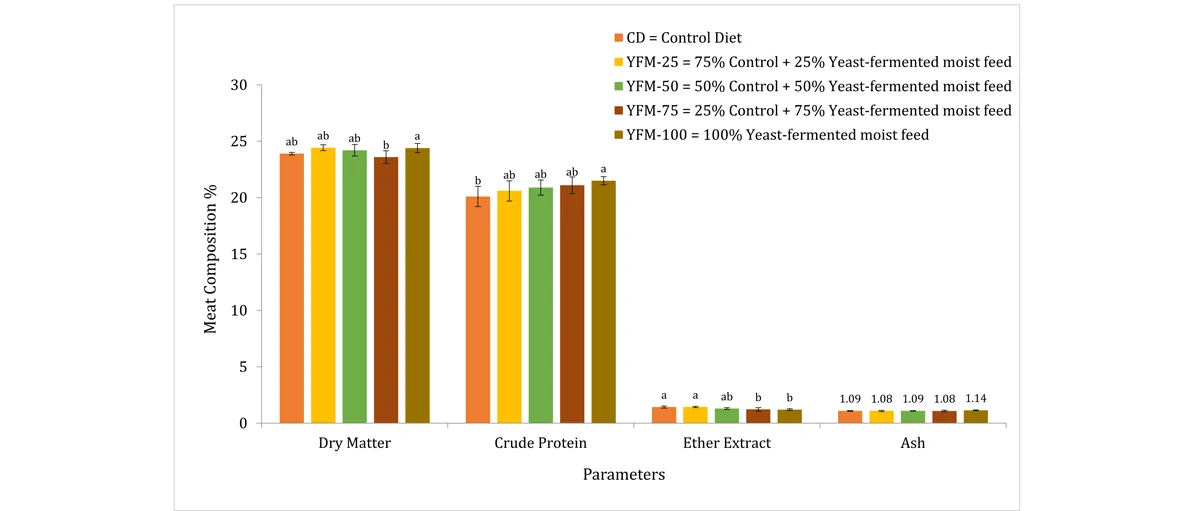
Meat composition of broilers fed yeast-fermented moist feed under different dietary treatments.

The nutritional composition of experimental broiler meat is shown in Figure 1 and indicates that the DM and CP percentages were significantly (*p* < 0.05) higher in the YFM-100 group than in the other dietary groups. DM percentage was lower (*p* < 0.05) in the YFM-75 group. The CP% was lower (*p* < 0.05) in the CD group than in all treatment groups. The highest (*p* < 0.05) EE percentage was found in the control group, while the lowest value was found in the YFM-100 and YFM-75 groups, respectively.

### 3.6. Nutrient digestibility

**Figure 2.**
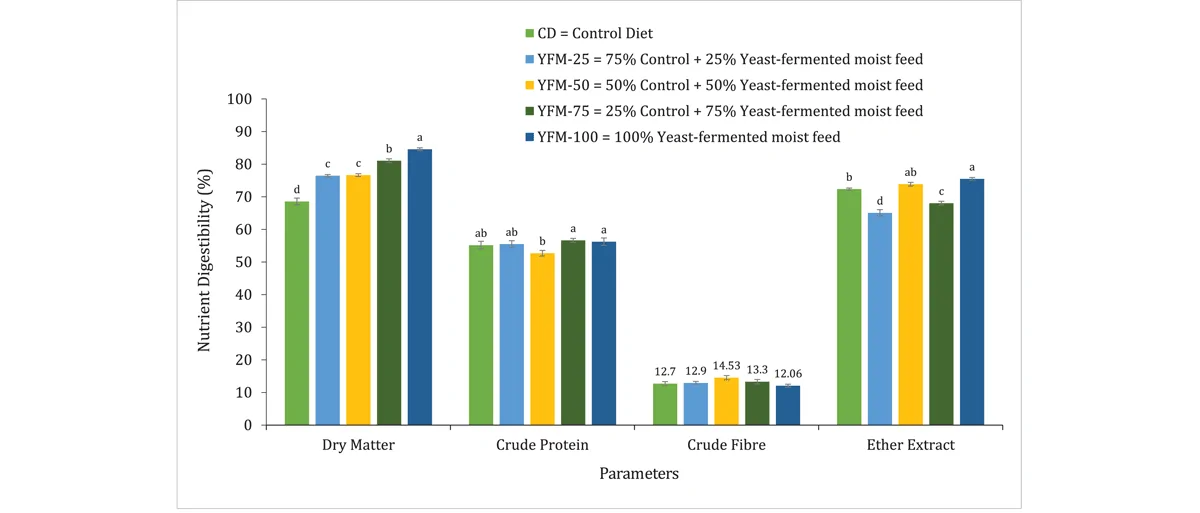
Nutrient digestibility of broilers fed yeast-fermented moist feed under different dietary treatments.

The nutrient digestibility of broilers fed yeast-fermented moist feed is shown in Figure 2, and it is stated that DM, CP, and EE digestibility percentages were significantly higher (*p* < 0.05) in the YFM-100 group, while DM digestibility percentage was lower in the CD group, and CP digestibility was lower in the YFM-50 group. CF digestibility percentage did not differ significantly (*p* > 0.05) among the treatment groups.

### 3.7. Tibia ash and bone mineralization

The tibia ash and the bone mineralization of experimental broilers are shown in Table 6. The tibia length, width, and weight were highest (*p* < 0.05) in the YFM-75 and YFM-100 compared to the control and other groups. The percentage of tibia ash was significantly higher (*p* < 0.005) in the YFM-100 (17.35 gm) and YFM-75 (17.19 gm) groups than in the control and other experimental groups.

**Table 6.** Tibia ash and bone mineralization of broiler chicken fed yeast-fermented moist feed under different dietary treatments.

Parameters	CD	YFM-25	YFM-50	YFM-75	YFM-100	SEM	*p*-value
Length (cm)	8.70 ± 0.11^b^	8.89 ± 0.18^b^	8.54 ± 0.37^b^	9.58 ± 0.33^a^	9.81 ± 0.28^a^	0.14	0.001
Width (cm)	0.76 ± 0.02^b^	0.84 ± 0.05^b^	0.86 ± 0.02^b^	1.04 ± 0.07^a^	1.07 ± 0.10^a^	0.03	0.001
Weight (gm)	9.04 ± 0.25^c^	9.50 ± 0.39^c^	9.56 ± 0.42^c^	11.38 ± 0.07a^b^	12.00 ± 0.22^a^	0.31	<0.001
ASH (%)	13.6 ± 0.59^b^	14.70 ± 0.58^b^	16.15 ± 0.89^ab^	17.19 ± 0.78^a^	17.35 ± 0.44^a^	0.42	<0.001

Here, CD = control diet; YFM-25 = 75% Control + 25% Yeast-fermented moist feed; YFM-50 = 50% Control + 50% Yeast-fermented moist feed; YFM-75 = 25% Control + 75% Yeast-fermented moist feed; YFM-100 = 100% Yeast-fermented moist feed; gm = gram; cm = centimeter; % = percentage; SEM = standard error of the mean; ^a,b,c,^ means bearing uncommon superscripts in a row differ significantly.

### 3.8. Cost-benefit ratio

Cost-benefit ratio of broilers fed yeast-fermented moist feeds are presented in Table 7. Feed cost and total production cost per kilogram of live weight of broiler were significantly higher (*p* < 0.001) in the control group than in the others. On the other hand, the profit per kilogram of live weight of the bird was significantly (*p* < 0.001) higher in the YFM-100 (0.31$) and the YFM-75 group (0.29$) compared to the control group (0.14$). The cost-benefit ratio was significantly (*p* < 0.001) higher in the YFM-100 (0.26) compared to the other dietary treatment groups.

**Table 7.** Cost-benefit ratio of broiler chicken fed yeast-fermented moist feed under different dietary treatments (USD).

Parameters (USD)	CD	YFM-25	YFM-50	YFM-75	YFM-100	SEM	*p*-value
Feed cost/kg LW	0.99 ± 0.01^a^	0.93 ± 0.01^ab^	0.89 ± 0.0.3^bc^	0.85 ± 0.01^c^	0.83 ± 0.02^c^	0.014	0.001
Total production cost /kg LW	1.36 ± 0.01^a^	1.30 ± 0.03^ab^	1.26 ± 0.03^bc^	1.21 ± 0.01^c^	1.20 ± 0.01^c^	0.014	0.001
Profit/kg LW	0.14 ± 0.02^c^	0.20 ± 0.02^bc^	0.25 ± 0.03^ab^	0.29 ± 0.01^a^	0.31 ± 0.03^a^	0.014	0.001
CBR	0.11 ± 0.00^c^	0.16 ± 0.01^b^	0.19 ± 0.02^bc^	0.24 ± 0.01^ab^	0.26 ± 0.01^a^	0.014	0.001

Here, CD = control diet; YFM-25 = 75% Control + 25% Yeast-fermented moist feed; YFM-50 = 50% Control + 50% Yeast-fermented moist feed; YFM-75 = 25% Control + 75% Yeast-fermented moist feed; YFM-100 = 100% Yeast-fermented moist feed; kg = kilogram; LW = live weight; CBR = cost-benefit ratio; USD = United States Dollar; SEM = standard error of the mean; ^a,b,c,^ means bearing uncommon superscripts in a row differ significantly; bird selling price = 190 tk/kg LW.

## 4. Discussion

### 4.1. Growth performance

Feed fermentation improved the nutritional value of animal feed ingredients [15, 17]. This study highlights the useful impacts of incorporating yeast-fermented moist feed on the growth performance of broiler chickens. Broilers fed a 75% or 100% fermented moist feed achieved the highest FBW, BWG, ADG, and improved FCR, while the control group exhibited the lowest growth performance. This progressive increase in growth performance with increasing fermentation levels suggests a dose-dependent positive effect of fermented moist feed on broiler performance. The existing results make parallels with numerous studies [29, 30, 31], which observed growth promotion in broiler chickens fed *S. cerevisiae*-fermented moist diets. This better growth performance is exhibited by various factors.

First of all, the yeast cell wall contains a significant amount of high-quality protein and non-starch polysaccharides such as β-glucan and mannan oligosaccharides (MOS) and small amounts of chitin [32]. These components improve intestinal health by increasing villus height, the ratio of crypt depth to villus height, ultimately improving nutrient absorption. Yeast (*S. cerevisiae*) helps maintain gut microbiota balance by suppressing pathogenic bacteria and increasing beneficial bacteria, thereby improving feed efficiency and growth performance in broilers [33]. Secondly, yeast itself is a better source of nutrients such as amino acids, B vitamins, minerals, and nucleotides, thereby increasing nutrient concentration for absorption. It also promotes the secretion of digestive enzymes, such as amylases, lipase, and protease, that improve the digestion of dietary nutrients.

### 4.2. Carcass characteristics

The dressing yield, head, shank, breast, thigh, and drumstick weights increased linearly with the inclusion level of yeast-fermented moist feed compared to other treatments, but gizzard, heart, and spleen weights were not significantly varied (*p* > 0.05) for all dietary treatment groups. These results line up with earlier studies demonstrating the significant influence of yeast supplementation on carcass yield [34, 35]. Some previous findings do not align with the current findings, stating that yeast supplementation does not have a significant effect on carcass characteristics [29, 36]. According to Ahmed et al. [37], yeast supplementation has no significant effect on carcass traits, whereas 3% yeast supplementation can reduce the liver weight compared to a control diet. Grigore et al. [38] found that yeast supplementation positively affects gizzard weight, which is inconsistent with our findings. This may be due to differences in yeast dose in the diet, bird strain, basal feed, or environmental factors.

### 4.3. Meat quality

Cooking loss, drip loss, and WHC are critical factors in meat quality, as they directly influence meat palatability and nutritional value. Higher inclusion levels of yeast-fermented moist feed (75% and 100%) reduced cooking and drip losses and increased water-holding capacity but had no significant effect on meat pH. The improved cooking loss and drip loss percentages are related to meat quality, resulting in more tender and juicier meat [39]. The observed improvement in WHC in the YFM-100 group suggests a potential enhancement in both the sensory and nutritional quality of the meat and may be related to muscle structural integrity and microbial modulation as previously reported by Hossain et al. [29]. Water loss during cooking not only affects the juiciness and tenderness of the meat but can also lead to a loss of essential nutrients [40]. The positive effects of dietary probiotics on meat quality observed in this study, particularly the improvements in cooking loss, drip loss, and WHC, are consistent with findings from several other studies [29, 34] that reported the significant positive impact on meat quality. After slaughter, the conversion of glycogen to lactic acid through glycolysis leads to a decline in muscle pH [41]. This pH drop is closely correlated with drip loss, a significant factor influencing meat quality as it reflects the muscle’s water-holding capacity [42]. These findings suggest that yeast addition might influence muscle glycolysis, thereby reducing lactic acid accumulation. Consequently, reduction in drip loss and cooking losses contributes to improved water-holding capacity, cooking yield, and overall meat quality.

### 4.4. Meat color

Meat color is an important parameter, as it influences consumer preferences. The impact of dietary treatments on meat color was evaluated in two distinct regions: breast and thigh. The lightness (L*) of meat was non-significant for both breast and thigh meat. The redness (a*) and yellowness (b*) were significant in yeast-fermented moist feed compared to the control in both breast and thigh meat. Meat color is related to pH because lower pH values are associated with rapid denaturation of meat proteins, which can cause pale meat and also reduce WHC [43]. So, here the increased WHC and lower protein denaturation might be related to the higher pH value in the yeast-supplemented group [44]. It is important to acknowledge that the effects of dietary interventions on meat color can vary with several factors, including the feeding stage, treatment type, and dosage. This could explain the observed differences in lightness and yellowness values between our study and those reported by Cheng et al. [45]. Future studies should aim to elucidate the specific mechanisms by which different dietary treatments influence meat color attributes. A comprehensive understanding of these mechanisms will enable the development of tailored feeding strategies to optimize meat quality and enhance consumer acceptability.

### 4.5. Meat composition

DM and CP contents were significantly higher in the major inclusion level of yeast-fermented moist feed than in the control. The ether extract content was higher in the control group but lower in the yeast-fermented group. This finding aligns with Hossain et al. [46], who reported higher crude fat content in the breast meat of broilers fed a diet containing 0.5% probiotics. The reduction in crude fat content with probiotic supplementation has also been reported in broiler breast meat [47]. The increase in DM content may reflect greater protein & mineral deposition in the meat. Adding fermented feed stabilizes muscle pH, improves membrane integrity, and reduces intracellular water content, finally increasing the DM percentage of meat. Therefore, the observed increase in CP content in the yeast-fermented groups was likely due to the microbial protein enrichment, more amino acid absorption & nitrogen retention, and ultimately increased muscle protein synthesis in broilers. The enhancement of fatty acid oxidation and a proportional increase in muscle protein synthesis might be the reason for the decrease in the EE content of meat in the fermented group.

### 4.6. Nutrient digestibility

The DM, CP, and EE digestibility were higher at the 100% inclusion level of fermented moist feed than in the control group at 35 days of age. The CF content did not differ significantly across the dietary treatment groups. These results are similar to those of Liu et al. [42], who reported significant differences in the DM digestibility of fed dry or wet feed in broilers. The enhanced nutrient digestibility observed with fermented feed could be attributed to microbial activity during fermentation. Furthermore, increased intestinal digestive enzyme activity, as observed in other studies with fermented feeds [48], contributes to higher feed utilization and absorption capacity. This study demonstrated a significantly positive impact of fermented moist feed on crude fiber efficiency during broiler metabolism compared with the control diet.

### 4.7. Tibia ash and bone mineralization

This study examined the impact of varying levels of yeast-fermented moist feed on broiler tibia characteristics. Results indicate a strong correlation between higher inclusion levels of fermented feed (75% and 100%) and improved bone quality indicators. These groups exhibited significantly greater tibia length, width, and weight compared to the control group, suggesting enhanced bone growth and development. These findings point out the potential of yeast-fermented moist feed, particularly at higher inclusion levels, to enhance bone health in broilers, likely due to improved nutrient availability and utilization [6]. The observed improvements in tibia characteristics can be attributed to the enhanced nutritional profile of the fermented feed. Fermentation has been shown to enhance the bioavailability of various essential minerals, including molybdenum, which plays a critical role in bone development and metabolism [49].

### 4.8. Cost-benefit ratio

This study examined the effects of incorporating yeast-fermented moist feed into the broiler diet on production costs and profitability. Feed cost and total production cost were significantly higher in the control group, whereas profit was higher in the YFM-100 group. The cost-benefit ratio was also higher in the group with the highest yeast-fermented moist feed inclusion than in the others. These findings align with existing research indicating that fermented feed ingredients, such as Fermented Dried Brewer’s Grain (FDBG), can reduce feed costs by 10% and improve broiler feed conversion ratios through the presence of probiotics [34].

## 5. Conclusions

The findings of current research indicate that a higher inclusion level of yeast-fermented moist feed can improve growth performance, nutrient digestibility, and bone mineralization, and enhance the meat quality of broiler chickens. Furthermore, the addition of yeast-fermented moist feed yields greater economic profit. The result suggests that completely fermented moist feed can enhance both farmer profitability and consumer satisfaction by producing safe meat. In the future, further research may examine blood metabolites and intestinal morphology to elucidate the beneficial effects of yeast-fermented moist feed on different broiler strains.

## Data Availability

The data presented in this study are available from the corresponding author upon reasonable request.

## List of abbreviations

AIA, acid insoluble ash; ANOVA, analysis of variance; ADG, average daily gain; ADF, acid detergent fiber; BWG, body weight gain; CF, crude fiber; CP, crude protein; Co., company; cm, centimeter; CRD, completely randomized design; DOC, day-old chick; DM, dry matter; DMRT, Duncan’s Multiple Range Test; EE, ether extract; FCR, feed conversion ratio; FBW, final body weight; FI, feed intake; FDBG, fermented dried brewer’s grain; gm, gram; mg, milligram; kg, kilogram; kcal., kilocalorie; ME, metabolic energy; ml, milliliter; NDF, neutral detergent fiber; NFE, Nitrogen-free extract; P, Phosphorus; h, hour; I.U., International units; WHC, water holding capacity; L*, Lightness; a*, Redness; b*, Yellowness; $, dollar; *, 5% level of significance; **, 1% level of significance; µg, microgram.
